# Advanced magnetic resonance imaging detects altered placental development in pregnancies affected by congenital heart disease

**DOI:** 10.21203/rs.3.rs-3873412/v1

**Published:** 2024-01-23

**Authors:** Daniel Cromb, Paddy Slator, Megan Hall, Anthony Price, Daniel Alexander, Serena Counsell, Jana Hutter

**Affiliations:** King’s College London; Cardiff University; King’s College London; King’s College London; University College London; King’s College London

## Abstract

Congenital heart disease (CHD) is the most common congenital malformation and is associated with adverse neurodevelopmental outcomes. The placenta is crucial for healthy fetal development and placental development is altered in pregnancy when the fetus has CHD. This study utilized advanced combined diffusion-relaxation MRI and a data-driven analysis technique to test the hypothesis that placental microstructure and perfusion are altered in CHD-affected pregnancies. 48 participants (36 controls, 12 CHD) underwent 67 MRI scans (50 control, 17 CHD). Significant differences in the weighting of two independent placental and uterine-wall tissue components were identified between the CHD and control groups (both p_FDR_<0.001), with changes most evident after 30 weeks gestation. A Significant trend over gestation in weighting for a third independent tissue component was also observed in the CHD cohort (R = 0.50, p_FDR_=0.04), but not in controls. These findings add to existing evidence that placental development is altered in CHD. The results may reflect alterations in placental perfusion or the changes in fetal-placental flow, villous structure and maturation that occur in CHD. Further research is needed to validate and better understand these findings and to understand the relationship between placental development, CHD, and its neurodevelopmental implications.

## Introduction

The placenta delivers oxygen and nutrients to the developing fetus, removes carbon dioxide and waste products, and performs a host of endocrine and immune functions. Placental development and function is key to healthy fetal development and impaired development can be linked to future health outcomes^1^, extending beyond the fetal and early neonatal period, into adulthood^2^ and can even affect future generations^3^.

Congenital heart disease (CHD) is the most common congenital malformation, affecting ~ 1% of live-births^4^, and is associated with impaired brain development and adverse neurodevelopmental outcomes, including motor, language and cognition,^5^ which persist into adulthood^6–8^. Previous studies suggest that placental development is altered in pregnancies where the fetus has CHD: placental weight and volume can differ, depending on the type of CHD^9,10^ and gross morphological changes, such as the insertion site of the umbilical cord, have been recorded^11,12^. Histological studies have identified differences^13,14^, including an increased incidence of thrombosis and infarction^15^ and vascular malperfusion lesions^16,17^. In certain CHD subtypes, placental gene expression and nutrient transfer is abnormal^18^ and MR imaging studies, although limited in number, have revealed differences in placental function^19,20^.

The fetal heart and placenta are both embryological fetal vascular organs, with shared expressed gene pathways^21,22^. It is plausible that placental vasculature may also be disrupted in fetal CHD, although the mechanisms behind exactly how remain unclear^23^. Recently, focus has shifted to the “heart-brain-placenta axis”^24–28^ to improve our understanding of the impaired brain and placental development seen in CHD. For example, there was a trend towards more severe brain injury in neonates with CHD when placental pathology was present^13^ and individuals with both CHD *and* placental abnormalities have Significantly lower cognitive and motor performance scores in early childhood^29^, although understanding the exact relationship between these factors is complex.

Identifying differences in structure and/or function of the CHD placenta is now a key area for research in the fetal CHD population^23,30^, with the hypothesis that altered placentation can result in decreased cerebral oxygen delivery and thus may be associated with impaired early brain development and subsequent adverse neurodevelopmental outcomes in CHD.

Placental histopathological examination is undoubtedly helpful for identifying gross morphological and microscopic changes in the CHD placenta^31^. However, placental histopathology has been likened to performing an autopsy, where important information only becomes available after delivery, and by which time placental structure and functional properties have changed substantially^32–34^. There is therefore a need for techniques to quantify placental health, structure and function in-utero.

Ultrasound, the current ‘gold-standard’ screening tool during pregnancy, is not suitable for assessing placental function or microstructure. The complex cascade of events, from inflow of highly oxygenated maternal blood through the spiral arteries, to exchange across the syncytiotrophoblast calls for comprehensive in-utero investigations exploring factors such as perfusion and tissue oxygenation. Placental MRI is a safe, non-invasive technique allowing such in-utero analysis, with both T2*-relaxation and diffusion imaging techniques used to assess placental function and microstructure throughout gestation^35–39^.

T2*-relaxation exploits the blood oxygen level dependent effect linking shorter T2* values to, amongst other factors, a higher concentration of deoxygenated hemoglobin, and is often interpreted as a proxy for placental oxygenation or function^40,41^. Since placental T2* is sensitive to the balance between oxygenated maternal blood delivery and fetal oxygen demand, it is particularly well-suited to identify altered placental oxygenation or blood flow patterns, as might be seen in CHD^20,39,41–45^.

Diffusion MRI is sensitive to the speed and directionality of motion of water molecules and thereby provides information relating to tissue microstructure. Importantly, the combination of an acquisition comprising multiple MR images and a mathematical model unlocks sensitivity to structures much smaller than the voxel size^46^.

Emerging combined diffusion-relaxation MRI techniques enable the acquisition of multi-modal diffusion and relaxation data in a single, efficient MR scan, instead of the conventional sequential scans. Measuring diffusion and relaxation simultaneously allows disentanglement of distinct tissue microenvironments that cannot be distinguished with T2*-relaxation or diffusion MRI alone^47,48^. Combined diffusion-relaxation MRI has been demonstrated in the placenta using the ZEBRA^49^ technique, and shows promise for identifying placental dysfunction^48^. Additionally, an unsupervised, data-driven analysis technique known as *InSpect* has been developed, enabling simultaneous assessment of placental oxygenation, microstructure and microcirculation from T2*-diffusion data^48,50^. Combining such advanced MRI acquisition and analysis techniques enables in-vivo investigation of multiple microstructural and perfusion environments, such as those found in the placenta.

This study utilized advanced combined diffusion-relaxation MRI and an unsupervised, data-driven analysis technique (InSpect) to test the hypothesis placental microstructure and perfusion are altered in-utero in pregnancies affected by CHD.

## Results

### Participant demographics

48 individual participants (36 controls, 12 CHD) satisfied the inclusion criteria, resulting in imaging data from 67 scans in total (50 control, 17 CHD). Maternal demographics for all participants are in Table 1. There was a Significant difference in maternal age at scan between the control and CHD samples, (median age 35.0 years (33.2–37.1) vs 32.3 years (27.9–34.9) respectively, p = 0.0079). There was no difference in scan GA or BMI between groups. Diagnoses included in the CHD cohort were: Coarctation of the aorta (CoA) = 4; Tetralogy of Fallot (ToF) = 1; Transposition of the Great Arteries (TGA) = 3; Hypoplastic Left Heart Syndrome (HLHS) = 3; Truncus Arteriosus (TA) = 1.

### Placental volume, T2* & ADC measurements

**Figure 1:**
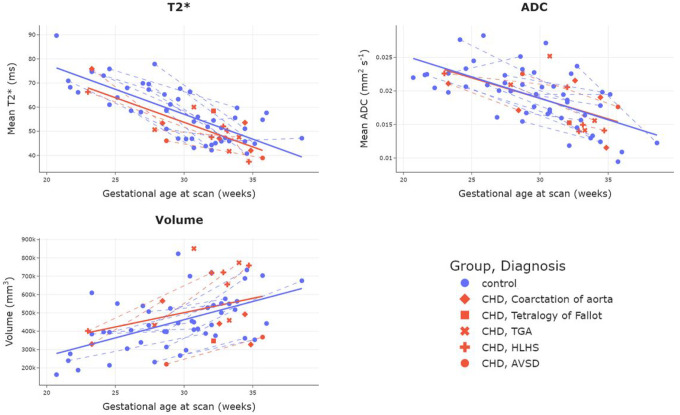
Plots showing mean placental and adjacent uterine wall T2* (top left), ADC (top right) and volume (bottom left) over gestation for all scans. Dotted lines join participants who had two scans.

Results of ROI volume, mean T2* and mean ADC values are shown in Table 2. After accounting for scan GA and maternal age, mean T2* was Significantly lower in the CHD sample (mean T2*: CHD 51.1 ± 9.9ms, Control 58.1 ± 11.4ms, p = 0.049), but there were no Significant differences in volume or mean ADC values between groups. Plots showing mean placental volume, T2* and ADC values across gestation for all 67 scans are shown in Fig. 1. Placental volume increased Significantly with GA (R = 0.50, p < 0.0001). Both mean T2* and ADC decreased Significantly with GA (R=−0.78, p < 0.0001; R=−0.63, p < 0.0001 respectively).

### Interpreting microenvironments

**Figure 2:**
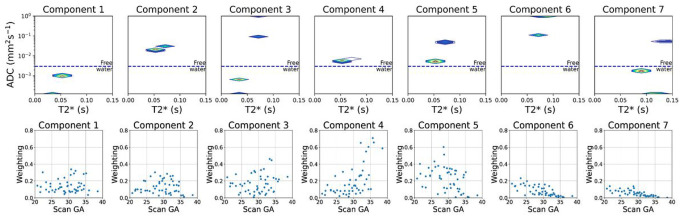
Seven component T2*-ADC spectra, determined by fitting the T2*-ADC model described in Equation 1 voxelwise to the whole placenta and uterine wall ROI, for data from 36 control participants (top-row). Plots showing the mean ROI signal weighting across gestation are shown on the bottom-row.

The derived T2*-ADC control spectra for each of the seven InSpect components, after running ‘full InSpect’ on data from 36 control participants, are shown in Fig. 2. Plots showing the mean MRI signal weighting for each ROI as a proportion of the total signal for each of the seven components across gestation, for all control participants, are also shown.

**Figure 3:**
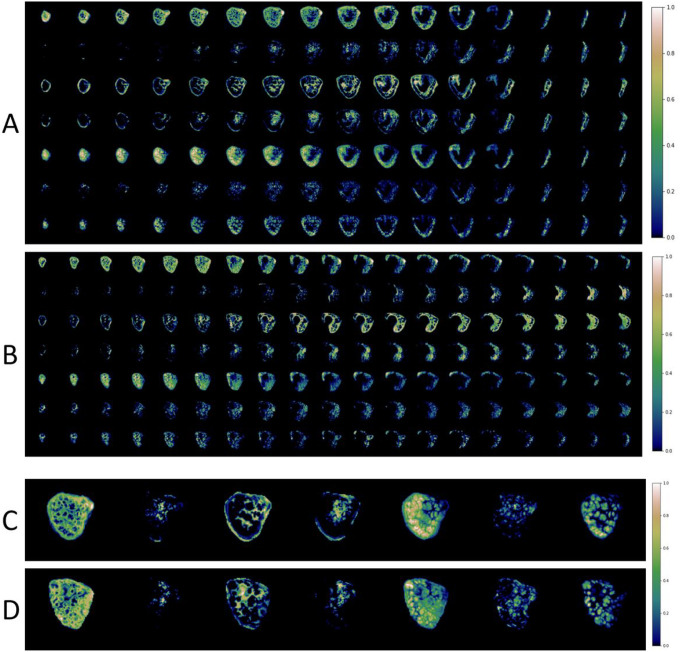
Whole placental composite images for two participants acquired at comparable gestational ages. Panel A is from a control participant, acquired at 34^+3^ weeks. Panel B is from a CHD participant, acquired at 34^+5^ weeks. The rows represent each component (1–7) and the columns represent slices through the ROI (left-to-right = anterior-to-posterior). Panel C and panel D show selected mid-placental slices from the same control (C) and CHD (D) dataset, highlighting the spatial location of each component at a gestational age of 34–35 weeks. The color scale (0 to 1) is the same for all images, representing the proportion of MR signal present in each voxel for each component.

Figure 3 shows composite spatial maps for both a control and a CHD participant, acquired at comparable GAs, highlighting differences in voxelwise weightings for all seven components (rows) and all placental slices (columns). Component one has two spectral peaks, both with relatively low T2* (< 0.06s) and ADC (< 0.001mm^2^s^− 1^) values, representing poorly oxygenated tissues with lower diffusivity. The spatial maps for this component show the highest signal in the periphery of placental lobules. Component three contains some spectral peaks with higher T2* (> 0.07s) and ADC (> 0.1mm^2^s^− 1^) values and, particularly at later gestations, is conspicuously absent from within placental lobules. It has a high signal towards the edge of the placenta and adjacent uterine wall and could be interpreted as representing connective tissue structures such as placental septa, as well as blood in vasculature within the uterine wall. Component seven has multiple spectral peaks, all with relatively high T2* values (> 0.09s), reflecting well oxygenated tissues. The spatial maps show it is confined within the placental lobules, with ‘hot-spots’ at the center of each lobule, and may therefore represent blood flowing into the lobules via uterine spiral arteries, before being slowed abruptly as it travels through the villous tree architecture at the fetal-maternal exchange surface.

An overview of the spectral peaks, mean signal-weighting contribution, how this weighting changes over gestation and the spatial distribution for each component, used for interpretation of the underlying tissue environments, are in *Supplementary Table 1*. Selected mid-placental slices for all participants for each component are in supplementary Figs. 1–7.

### Component weighting plots

**Figure 4:**
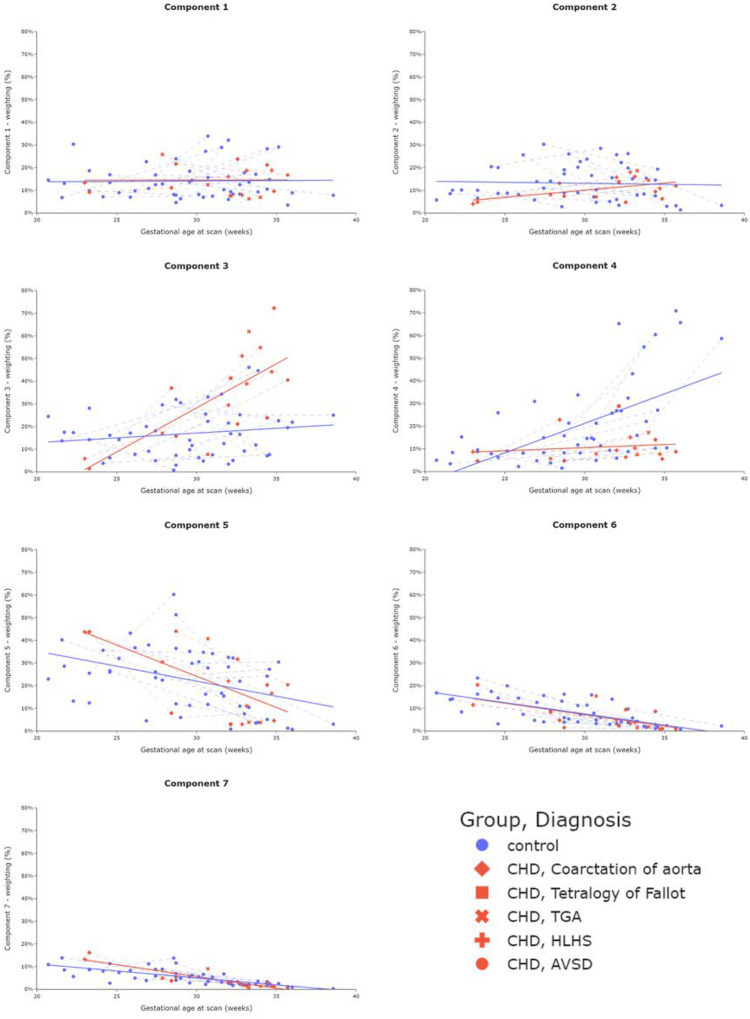
Mean ROI (placenta and adjacent uterine wall) component weightings across gestation from 67 scans (50 control, 17 CHD). Component weighting ranges (y-axis) are kept consistent (0–80%) to aid interpretation as to the overall contribution to the MR signal from each component. Dotted lines join participants who underwent two scans.

All component weighting plots are shown in Fig. 4.

After accounting for GA at scan and maternal age, there was a Significant difference in mean ROI weightings between control and CHD groups for component three and component four (both p_FDR_<0.001).

For control data, component four was the only component to show a Significant increase across gestation, occurring most noticeably after 30 weeks (R = 0.60, p_FDR_<0.001). Components five, six and seven show a Significant decrease (R=−0.40, p_FDR_=0.004; R=−0.71, p_FDR_<0.001; R=−0.74, p_FDR_<0.001 respectively).

For CHD data, components two and three showed a Significant increase across gestation (R = 0.50, p_FDR_=0.040; R = 0.73, p_FDR_=0.0013 respectively), whereas components five, six and seven show a Significant decrease (R=−0.67, p_FDR_=0.0033; R=−0.68, p_FDR_=0.0025; R=−0.89, p_FDR_<0.001 respectively)

## Discussion

This is the first study utilizing combined diffusion-relaxation MRI to explore placental structure and function in-utero in CHD-affected pregnancies. We used a data-driven approach simultaneously sensitive to oxygenation, microstructure and microcirculation^50^ to show that independently derived placental and adjacent uterine wall tissue environments change Significantly during key periods of fetal development, between 20 and 40 weeks gestation, in both normal pregnancies and those where the fetus has CHD.

For multiple components, different trends over gestation for control and CHD data are observed (Fig. 4). The weighting of component three increases noticeably after 30 weeks in CHD cases, in contrast to the control sample. For component four, the increase in weighting after 30 weeks in control participants is not reflected in the CHD data. Based on their MR properties and spatial distribution (see *Supplementary Table 1*), component three could represent poorly perfused structures such as placental septa, as well as blood within vasculature in the uterine wall and component four may represent blood returning from the fetus and draining into maternal veins. Importantly, however, this result is independent of any interpretation of the specific placental microenvironments these components represent. A Significant trend over gestation in weighting for an additional independently-derived component was also observed in the CHD cohort, but not in the control cohort.

These findings add to existing evidence that placental development is altered in CHD, and complements research using combined diffusion-relaxation MRI to identify placental compartments with distinct T2*-ADC combinations^51^ and abnormal placentation associated with pregnancy-related conditions PE or FGR^48,52^. The results reported here have the potential to help with understanding of the interlinked pathways between placental and cardiac development^22^.

One hypothesis is that changes observed here may reflect alterations in placental perfusion seen in CHD^43,53^. However, given the small difference in mean placental T2* between groups, and the difference in trajectories between T2* and the weighting of components three and four over gestation, it is unlikely that reduced perfusion alone is driving these differences. Changes in fetal-placental flow^54^ and villous structure^18^ that occur in CHD may also be contributing. As pregnancy advances, specific microstructural changes occur within the placenta, particularly in the third trimester^55^, including terminal villi development^56^ and fetal villous angiogenesis^57^. This is an important adaptation that ensures efficient oxygen and nutrient exchange in the later stages of gestation, to meet fetal demands, but as terminal villous development is directly influenced by placental oxygen levels in normal pregnancy^58^, this process may be altered in CHD. Altered villous maturation, consistent with an ‘immature’ placental microvasculature, could also be preventing maximal oxygenation of fetal blood in CHD^23^. It is interesting to note that the GA after which the differences in the weighting of components three and four between groups becomes most apparent − 30 weeks - is consistent with the GA at which volumetric brain development also deviates from normal in fetuses with CHD^59^.

**Figure 5:**
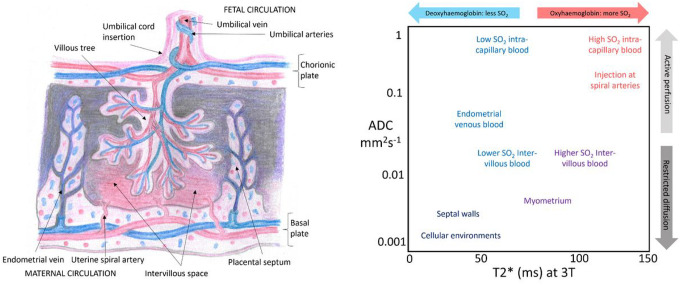
Placental schematic depicting placental anatomy and structures at ~32 weeks gestation (left), and the corresponding speculative tissue environments characterized by their T2*-ADC properties (right).

The approach we have used involves no a-priori understanding of different placental compartments or microstructural environments, but identifies them based on shared T2*-ADC characteristics and an understanding of placental structure (Fig. 5). The fetal circulation is intra-capillary and has a relatively low oxygen saturation at the exchange surface, whereas the maternal circulation enters the placenta as a highly-saturated blood pool, but is extravascular in the human placenta^47^. Despite this complex, heterogeneous structure, previous studies have used mean whole-placental MRI biomarkers^60,61^, making interpretation of the results challenging. Identifying unique placental tissue compartments that might be altered in CHD fits the inherent complexity of the placenta and thus helps provide a focus for future research studies. The associated spatial maps aid in localisation of these compartments, helping differentiate tissue environments as they change throughout the placenta, i.e. from basal to decidual plate.

Additionally, the complexity of placental physiology benefits from a comprehensive assessment approach, such as this combined use of diffusion-relaxation and a bespoke analysis tool like InSpect. Whilst a small but Significant decrease in whole placental T2* was identified in the CHD cohort, there was no Significant difference in whole placental ADC values between groups. This further highlights the enhanced sensitivity of InSpect to detect changes in placental function and microstructure beyond the use of T2* or ADC independently.

However, it is important to emphasize that using a data-driven approach means components don’t have to neatly define placental compartments such as “fetal” and “maternal”, or “intracellular” and “extracellular”, but can also reflect combined tissue environments. Placental anatomy means tissue environments with different T2*-ADC properties can sit in close proximity, i.e. at the villous exchange surface, where pooled maternal blood lies close to fast flowing fetal blood in small diameter vessels, or at the umbilical cord insertion site, where similar sized vessels containing blood with very different T2* properties intertwine.

It is also worth highlighting that InSpect provides the *proportion* or *weighting* of the MR signal in each voxel that each component represents. This results in component weightings that are intrinsically linked, and may explain why there appears to be such a ‘reciprocal’ change in components 3 and 4, since a reduction in the weighting for one component necessitates an increase in another. This makes it challenging to interpret whether both components are affected, or just one, and this might reflect underlying changes in placental microstructure or perfusion. However, this result is independent of any interpretation of the specific placental microenvironments and suggests a clear difference in at least one compartment. Furthermore, any changes in component weighting, i.e. those seen over gestation, or between groups, suggests that the tissue environment(s) represented by that component change *as a proportion of total placental volume* over gestation, and not necessarily that there is a change in *absolute* volume.

As expected, we show that placental volume increases with advancing gestation. Consistent with previous work, we also identify a decrease in both T2* and ADC values with advancing gestations^39,40,62^, that appear consistent in healthy participants undergoing two scans^63^.

This study is limited by relatively small numbers at early gestations. The CHD cohort is also diagnostically heterogenous, and different diagnoses may impact placental development or function in different ways^13,15^. However, all CHD diagnoses were critical or serious, which is where the greatest alterations in placental development in CHD might be expected^12,64^. Different types of CHD may affect fetal-placental flows in different ways^43,65,66^, or involve different genes linking placental and vascular development^18^, so future work with larger cohorts is needed to explore how certain CHD diagnoses or physiologies might be associated with impaired placental development.

Using a data-driven technique such as InSpect involves speculation as to the underlying tissue environments or microstructures represented. There is currently no ‘ground truth’. We also did not collect ultrasound information, such as that relating to uterine artery resistance or dopplers, which may be helpful for establishing the presence of uteroplacental dysfunction^43^. Future work should involve attempts to validate and better understand these findings, either through histopathological examination, by invasive sampling^67^ or in comparison with complementary ultrasound techniques^68^.

Future work including data from other cohorts where placental dysfunction is better understood/characterised, for comparison to both CHD and control placentas, would also be beneficial. For example, others have previously identified associations between fetal CHD and maternal hypertensive disorders of pregnancy^69,70^, including the risk of pre-eclampsia^16^, hinting at a potential common etiology, which could be explored using this approach in future.

Furthermore, we did not collect data related to maternal or fetal haematinics. However, it is plausible that levels of fetal or maternal haemoglobin influence placental T2* values. Given that maternal haemoglobin levels change over gestation^71^, and that fetal haematinics are affected by both impaired placentation and CHD^72,73^, future work should attempt to capture and include this information.

The trends over GA of several component weightings, seen in both CHD and control placentas, could reflect normal changes in the microstructure the placenta as pregnancy advances^31,32,74^, with the corresponding differences in tissue microstructure and perfusion that occur^75^, and in future could serve as imaging biomarkers of both normal and abnormal placental development. Given enough data from typically developing placentas, InSpect could also be used to generate a quantitative ‘placental abnormality’ score, taking into account the deviation from normal of each component weighting for a given GA. This would enable both quantifiable analysis of impaired placentation, and help identify where within the placenta this occurs.

## Conclusions

We report using combined diffusion-relaxation MRI and a data-driven approach to detect altered placental tissue environments in pregnancies affected by fetal CHD, with changes most evident after 30 weeks gestation. We speculate that these changes are driven by impaired perfusion and microstructure in the CHD placenta, although future work is needed to definitively link these imaging findings to potential alterations in the underlying placental structure and function.

## Materials and Methods

### Ethics and Recruitment

Data were acquired as part of The Congenital Heart Disease Imaging Programme (CHIP) at St. Thomas’ Hospital in London. All methods were carried out in accordance with relevant guidelines and regulations and all experimental protocols were approved by a named institutional and/or licensing committee [NHS REC 21/WA/0075]. Control participants experiencing a low-risk pregnancy, with the absence of pregnancy-induced hypertension (PIH), preeclampsia (PE), fetal growth restriction (FGR), or gestational diabetes (GD) at the time of enrolment, were recruited after their antenatal booking or screening appointments. Participants with a fetus with severe or critical CHD, as defined previously^76^, confirmed on fetal echocardiography, were recruited from the fetal cardiology clinic. Participants with PIH, PE, FGR, GD, or where the fetus had confirmed genetic abnormalities were also excluded from the CHD cohort. All participants were invited to have up to two fetal MRI scans.

Data were subsequently excluded if the pregnancy resulted in a delivery before 37 weeks gestational age (GA), if PIH, PE, FGR or GD were newly diagnosed between scan and delivery, if any genetic abnormalities were detected on antenatal testing, or if any Significant incidental fetal or placental findings were reported on imaging. Data sets with insufficient quality, including cropping of the placenta, extensive geometric distortion artifacts, or visible contractions during the scan were also excluded.

### Image acquisition and reconstruction

Informed, written consent was obtained from all subjects prior to imaging. Images were acquired on a Philips Achieva 3T scanner using a 32-channel surface coil. All imaging was performed in supine position with frequent verbal interaction, continuous heart rate and oxygen saturation monitoring, and blood-pressure measurements at ten minute intervals.

Following a pilot scan and B0 and B1 calibration scans, anatomical imaging using T2-weighted turbo-spin-echo sequences, as well as a multi-echo gradient-echo sequence, a combined T2*-diffusion scan (ZEBRA^49^) was performed, with parameters defined in Table 3. Acquisition time for this sequence was 8m30s.

The acquired data were then anonymised and reconstructed using in-house tools, including denoising and motion-correction, as previously described^49^. The reproducibility of this T2*-diffusion sequence has been demonstrated in MR phantom, adult brain, and placental studies^49,63^.

### T2* and ADC mapping

**Figure 6:**
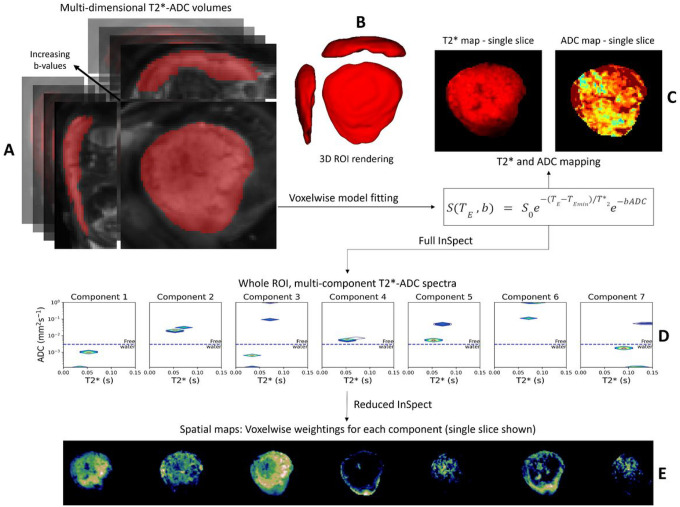
Data flow: Manual regions of interest (ROI) (placental and adjacent uterine wall mask) overlaid on the first multi-dimensional volume (b=0, TE=78ms) of the acquired diffusion-relaxation data (A), with the corresponding 3D-ROI rendering adjacent (B). After fitting equation 1 voxelwise to all voxels in this 3DROI, T2* and ADC maps were generated. These are shown for a selected coronal slice from a healthy placenta, acquired at 28^+4^ weeks gestation (C). Subsequently, ‘full’ InSpect was run on data from 36 control participants, to generate the T2*-ADC spectra associated with each of the seven components (D), and also calculate the voxelwise weightings of each component, shown here for the same selected coronal slice (E). These spectra were then used to infer voxelwise spatial maps for all CHD scans and additional ‘repeat’ scans from the control sample (‘reduced InSpect’).

Figure 6 outlines the data-processing workflow. First, a region of interest (ROI) containing the whole placenta and adjacent uterine wall section was manually segmented on the anonymised first b = 0 image with the lowest echo time, by an experienced clinician, who was blinded to the maternal demographics and fetal diagnosis. The T2*-ADC model described in Eq. 1 was then fit voxelwise using a modified version of the diffusion microstructure imaging in python toolbox^77^, as described in^63^, enabling calculation of the mean T2* and ADC values for the whole ROI.

### Data-driven analysis with InSpect:

We ran InSpect on the first scans from all 36 control participants using the InSpect toolbox (https://github.com/PaddySlator/inspect) (Fig. 6). Seven InSpect components were fixed as this number has previously been shown to best explain the placental and adjacent uterine-wall T2*-diffusion signal^50^. This full InSpect is a process akin to independent component analysis, albeit under the assumption that the data is generated by the underlying dynamics of Eq. 1. Full InSpect hence identified seven components in the data, with each component having a corresponding T2*-ADC spectra (e.g. Figure 6). The spectral peaks in these T2*-ADC spectra represent different tissue microenvironments within the ROI. InSpect has no a-priori information about the tissue or organ being imaged. For placental imaging, this full InSpect analysis is analogous to a ‘reverse-recipe’: it takes T2*-ADC data as input, and infers information about the unique tissue microenvironments (or components) that are required to ‘make’ each placenta as the output. The relative weighting of each of these components is calculated voxelwise during this full InSpect process, allowing maps quantifying the spatial distribution and relative amount of each component present in every voxel to be created (e.g. Figure 6). These components and their corresponding T2*-ADC spectral peaks in data from control participants were assumed to be representative of typically developing placentas. We then quantified how the relative fractions of these components change over GA.

Equation 1: $$S\left(T_{E},b\right)=S_{0}e^{-\left(T_{E}-T_{\text{Emin}}\right)/T_{2}^{*}}e^{-\text{bADC}}$$

where S_0_ is the signal at the proton density (b=0), T_E_ is the echo time, T_E_ is the shortest echo time acquired, is the effective transverse-relaxation time, *b* is the b-value and ADC is the apparent diffusion coefficient.

These spectra were then used to infer voxelwise spatial maps for all CHD scans and additional ‘repeat’ control scans, in a process we term ‘reduced InSpect’. Reduced InSpect fixes the values of the T2*-ADC spectra associated with each component, only calculating the corresponding maps, ensuring the components are identical for all data being analyzed with the additional benefit of computational efficiency. Continuing the previous analogy, this corresponds to using ‘reduced InSpect’ to quantify the proportions of each of these predefined components in each individual placenta. The results of these analyses were used to determine how much each component differs from normal for each CHD dataset.

### Interpreting microenvironments and plotting component weightings

Next, the overall contribution, or weighting, of each component of the InSpect analysis was plotted against GA for each dataset. The MR tissue properties of each component were then assessed, taking into account their T2*-ADC spectral peaks, with relatively higher T2* values representing more well-oxygenated tissue and higher ADC values (above free-water = 0.3mm^2^s^− 1^) representing perfusing or fast-flowing blood, usually interpreted as within vasculature. Combining this information with the MR signal weighting contributed by each component, how the weightings change over gestation, and how the components are spatially distributed, enabled speculations about the tissue microenvironments encoded by each component to be made.

### Statistical analyses

A Shapiro-Wilk test was used to test normality. An ANCOVA was used to compare placental volume, T2*, ADC and InSpect derived component-weightings between groups, after accounting for gestational age at scan and maternal demographics. Pearson’s correlation coefficient was calculated to determine the direction and strength of trends between continuous variables across the GA ranges studied. For analyses of component weightings, Benjamini and Hochberg false discovery rate (FDR) was applied to correct for multiple comparisons (reported as P_FDR_). P_FDR_-values < 0.05 were considered Significant. All statistical analyses were performed using statsmodels v0.13.2^78^ and Jupyter Notebook, python3.

## Figures and Tables

**Table 1:** Maternal participant demographics

Cohort	Participants	Scans	GA at scan (weeks)*	P^✝^	Maternal BMI at scan (kg/m^2^)*	P^✝^	Maternal age at scan (y)*	P^✝^
Control	36	50	29.9 (26.9–32.6)	-	26.2 (± 3.0)	-	35.0 (33.2–37.1)	-
CHD	12	17	32.6 (28.7–34.0)	0.09	26.3 (± 4.0)	0.91	32.3 (27.9–34.9)	**0.008**
*All*	*48*	*67*	*30.7 (27.4–33.1)*	-	*26.2 (± 3.0)*	-	*34.6 (32.5–36.7)*	-

* Values given are mean (± standard deviation) for parametric data, or median (25th centile-75th centile) for non-parametric data

✝ P-value for comparison between mean or median values for control and CHD cohort and control and PIH cohort separately. Results in **bold** are considered Significant.

**Table 2:** MRI derived placental characteristics

Cohort	Placental volume (mm^3^)*	P^✝^	Mean placental T2* (ms)	P^✝^	Mean placental ADC	P^✝^
Control	453,000 (± 152,000)	-	58 (47–67)	-	0.020 (0.016–0.022)	-
CHD	512,000 (± 190,000)	0.37	51 (46–54)	**0.49**	0.017 (0.014–0.021)	0.97
All	471,000 (± 163,000)	-	54 (47–65)	-	0.019 (0.016–0.022)	-

* Values given are mean (± standard deviation) for parametric data, or median (25th centile-75th centile) for non-parametric data

✝ P-value from ANCOVA between control and CHD cohort, after accounting for gestational age at scan and maternal age. Results in **bold** are considered Significant.

**Table 3:** Combined T2*-ADC multi-echo gradient-echo MRI scan acquisition parameters

Orientation: Coronal plane to maternal habitus, FOV = 300×320×84mm, Resolution: 3mm^3^ isotropic.
Echo Time = (78, 114, 150, 186) ms, Repetition Time = 7.5 ms, SENSE factor = 2.5.
b = (5, 10, 25, 50, 100, 200, 400, 600, 1200, 1600) s mm^−2^; 3 directions
b = 18 s mm^−2^; 8 directions
b = 36 s mm^−2^; 7 directions
b = 800 s mm^−2^; 15 directions

## Data Availability

The datasets analysed during the current study are available from the corresponding author on reasonable request

## References

[1] Heazell A. The placenta and adverse pregnancy outcomes – opening the black box? BMC Pregnancy Childbirth 15(Suppl 1), (2015).

[2] Konkel L. Lasting Impact of an Ephemeral Organ: The Role of the Placenta in Fetal Programming. Environ. Health Perspect. 124, A124–A129 (2016). 27479992 10.1289/ehp.124-A124 PMC4937843

[3] Roseboom T. J. & Watson E. D. The next generation of disease risk: Are the effects of prenatal nutrition transmitted across generations? Evidence from animal and human studies. Placenta 33, e40–e44 (2012). 22902003 10.1016/j.placenta.2012.07.018

[4] EUROCAT. European Platform on Rare Disease Registration. (2020).

[5] Latal B. Neurodevelopmental Outcomes of the Child with Congenital Heart Disease. Clin. Perinatol. 43, 173–185 (2016). 26876129 10.1016/j.clp.2015.11.012

[6] Ilardi D., Ono K. E., McCartney R., Book W. & Stringer A. Y. Neurocognitive functioning in adults with congenital heart disease. Congenit. Heart Dis. 12, 166–173 (2017). 27957813 10.1111/chd.12434

[7] Klouda L., Franklin W. J., Saraf A., Parekh D. R. & Schwartz D. D. Neurocognitive and executive functioning in adult survivors of congenital heart disease. Congenit. Heart Dis. 12, 91–98 (2017). 27650247 10.1111/chd.12409

[8] Rodriguez C. P., Clay E., Jakkam R., Gauvreau K. & Gurvitz M. Cognitive impairment in adult CHD survivors: A pilot study. Int. J. Cardiol. Congenit. Heart Dis. 6, 100290 (2021). 35425931 10.1016/j.ijcchd.2021.100290 PMC9006779

[9] Andescavage N. 3-D volumetric MRI evaluation of the placenta in fetuses with complex congenital heart disease. Placenta 36, 1024–1030 (2015). 26190037 10.1016/j.placenta.2015.06.013 PMC4554892

[10] Matthiesen N. B. Congenital Heart Defects and Indices of Placental and Fetal Growth in a Nationwide Study of 924 422 Liveborn Infants. Circulation 134, 1546–1556 (2016). 27742737 10.1161/CIRCULATIONAHA.116.021793

[11] Albalawi A. Placental Characteristics of Fetuses With Congenital Heart Disease. J. Ultrasound Med. 36, 965–972 (2017). 28258617 10.7863/ultra.16.04023

[12] Snoep M. C. Placenta morphology and biomarkers in pregnancies with congenital heart disease – A systematic review. Placenta 112, 189–196 (2021). 34388551 10.1016/j.placenta.2021.07.297

[13] Schlatterer S. D. Placental Pathology and Neuroimaging Correlates in Neonates with Congenital Heart Disease. Sci. Rep. 9, 4137 (2019). 30858514 10.1038/s41598-019-40894-y PMC6411739

[14] Leon R. L. Placental vascular malperfusion lesions in fetal congenital heart disease. Am. J. Obstet. Gynecol. S0002–9378(22)00389–1 (2022) doi:10.1016/j.ajog.2022.05.038. PMC13217299 35609643

[15] Rychik J. Characterization of the Placenta in the Newborn with Congenital Heart Disease: Distinctions Based on Type of Cardiac Malformation. Pediatr. Cardiol. 39, 1165–1171 (2018). 29728721 10.1007/s00246-018-1876-x PMC6096845

[16] Miremberg H. The association between severe fetal congenital heart defects and placental vascular malperfusion lesions. Prenat. Diagn. 39, 962–967 (2019). 31254468 10.1002/pd.5515

[17] O’Hare C. B. Placental Delayed Villous Maturation is Associated with Fetal Congenital Heart Disease. Am. J. Obstet. Gynecol. 0, (2022). 10.1016/j.ajog.2022.08.013 PMC10436378 35985515

[18] Courtney J. Abnormalities of placental development and function are associated with the different fetal growth patterns of hypoplastic left heart syndrome and transposition of the great arteries. Placenta 101, 57–65 (2020). 32927345 10.1016/j.placenta.2020.09.007

[19] Sun L. Reduced Fetal Cerebral Oxygen Consumption is Associated With Smaller Brain Size in Fetuses With Congenital Heart Disease. Circulation 131, 1313–1323 (2015). 25762062 10.1161/CIRCULATIONAHA.114.013051 PMC4398654

[20] Steinweg J. K. T2* placental MRI in pregnancies complicated with fetal congenital heart disease. Placenta 108, 23–31 (2021). 33798991 10.1016/j.placenta.2021.02.015 PMC7611398

[21] Wilson R. L. Analysis of commonly expressed genes between first trimester fetal heart and placenta cell types in the context of congenital heart disease. Sci. Rep. 12, 10756 (2022). 35750800 10.1038/s41598-022-14955-8 PMC9232495

[22] Mahadevan A., Tipler A. & Jones H. Shared developmental pathways of the placenta and fetal heart. Placenta 141, 35–42 (2023). 36604258 10.1016/j.placenta.2022.12.006

[23] Leon R. L. Neuroplacentology in congenital heart disease: placental connections to neurodevelopmental outcomes. Pediatr. Res. 1–8 (2021) doi:10.1038/s41390-021-01521-7. PMC9064799 33864014

[24] Huhta J. & Linask K. K. Environmental origins of congenital heart disease: the heart-placenta connection. Semin. Fetal. Neonatal Med. 18, 245–250 (2013). 23751925 10.1016/j.siny.2013.05.003

[25] Linask K. K. The heart-placenta axis in the first month of pregnancy: induction and prevention of cardiovascular birth defects. J. Pregnancy 2013, 320413 (2013). 23691322 10.1155/2013/320413 PMC3652177

[26] Camm E. J., Botting K. J. & Sferruzzi-Perri A. N. Near to One’s Heart: The Intimate Relationship Between the Placenta and Fetal Heart. Front. Physiol. 9, (2018). 10.3389/fphys.2018.00629 PMC6029139 29997513

[27] Cohen J. A., Rychik J. & Savla J. J. The placenta as the window to congenital heart disease. Curr. Opin. Cardiol. 36, 56–60 (2021). 33074934 10.1097/HCO.0000000000000816

[28] Peyvandi S. & Rollins C. Fetal Brain Development in Congenital Heart Disease. Can. J. Cardiol. 39, 115–122 (2023). 36174913 10.1016/j.cjca.2022.09.020 PMC9905309

[29] Segar D. E. The Relationship Between Placental Pathology and Neurodevelopmental Outcomes in Complex Congenital Heart Disease. Pediatr. Cardiol. (2022) doi:10.1007/s00246-022-03018-4. 36201029

[30] Snoep M. C. Factors Related to Fetal Demise in cases with Congenital Heart Defects. Am. J. Obstet. Gynecol. MFM 0, (2023). 10.1016/j.ajogmf.2023.101023 37220848

[31] Guttmacher A. E., Maddox Y. T. & Spong C. Y. The Human Placenta Project: placental structure, development, and function in real time. Placenta 35, 303–304 (2014). 24661567 10.1016/j.placenta.2014.02.012 PMC3999347

[32] Huppertz B. The anatomy of the normal placenta. J. Clin. Pathol. 61, 1296–1302 (2008). 18755720 10.1136/jcp.2008.055277

[33] Burton G. J. & Fowden A. L. The placenta: a multifaceted, transient organ. Philos. Trans. R. Soc. B Biol. Sci. 370, 20140066 (2015). 10.1098/rstb.2014.0066 PMC4305167 25602070

[34] Nelson D. M. & Myatt L. The Human Placenta in Health and Disease. Obstet. Gynecol. Clin. North Am. 47, xv–xviii (2020). 10.1016/j.ogc.2020.01.001 32008676

[35] Hutter J. Multi-modal functional MRI to explore placental function over gestation. Magn. Reson. Med. 81, 1191–1204 (2019). 30242899 10.1002/mrm.27447 PMC6585747

[36] Melbourne A. On the use of multicompartment models of diffusion and relaxation for placental imaging. Placenta 112, 197–203 (2021). 34392172 10.1016/j.placenta.2021.07.302

[37] Hansen D. N. T2*-weighted placental magnetic resonance imaging: a biomarker of placental dysfunction in small-for-gestational-age pregnancies. Am. J. Obstet. Gynecol. MFM 4, 100578 (2022). 35114424 10.1016/j.ajogmf.2022.100578

[38] Malmberg M. Perfusion fraction derived from IVIM analysis of diffusion-weighted MRI in the assessment of placental vascular malperfusion antenatally. Placenta 119, 1–7 (2022). 35066306 10.1016/j.placenta.2022.01.005

[39] Schabel M. C. Quantitative longitudinal T2* mapping for assessing placental function and association with adverse pregnancy outcomes across gestation. PloS One 17, e0270360 (2022). 35853003 10.1371/journal.pone.0270360 PMC9295947

[40] Hutter J. T2* relaxometry to characterize normal placental development over gestation in-vivo at 3T. Wellcome Open Res. 4, 166 (2019).

[41] Sørensen A., Hutter J., Seed M., Grant P. E. & Gowland P. T2*-weighted placental MRI: basic research tool or emerging clinical test for placental dysfunction? Ultrasound Obstet. Gynecol. 55, 293–302 (2020). 31452271 10.1002/uog.20855

[42] Schabel M. C. Functional Imaging of the Non-Human Primate Placenta With Endogenous BOLD Contrast. Magn. Reson. Med. 76, 1551–1562 (2016). 26599502 10.1002/mrm.26052 PMC4879634

[43] Binder J. Evidence for uteroplacental malperfusion in fetuses with major congenital heart defects. PLoS ONE 15, e0226741 (2020). 32023263 10.1371/journal.pone.0226741 PMC7001956

[44] Sørensen A. & Sinding M. Placental Magnetic Resonance Imaging: A Method to Evaluate Placental Function In Vivo. Obstet. Gynecol. Clin. North Am. 47, 197–213 (2020). 32008669 10.1016/j.ogc.2019.10.009

[45] Pan M. & Li D.-Z. Placenta insufficiency and congenital heart defects. Am. J. Obstet. Gynecol. MFM 101070 (2023) doi:10.1016/j.ajogmf.2023.101070. 37406988

[46] Bihan D. L. MR imaging of intravoxel incoherent motions: application to diffusion and perfusion in neurologic disorders. Radiology (1986) doi:10.1148/radiology.161.2.3763909. 3763909

[47] Melbourne A. Separating fetal and maternal placenta circulations using multiparametric MRI. Magn. Reson. Med. 81, 350–361 (2019). 30239036 10.1002/mrm.27406 PMC6282748

[48] Slator P. J. Combined diffusion-relaxometry MRI to identify dysfunction in the human placenta. Magn. Reson. Med. 82, 95–106 (2019). 30883915 10.1002/mrm.27733 PMC6519240

[49] Hutter J. Integrated and efficient diffusion-relaxometry using ZEBRA. Sci. Rep. 8, 15138 (2018). 30310108 10.1038/s41598-018-33463-2 PMC6181938

[50] Slator P. J. Data-Driven multi-Contrast spectral microstructure imaging with InSpect: INtegrated SPECTral component estimation and mapping. Med. Image Anal. 71, 102045 (2021). 33934005 10.1016/j.media.2021.102045 PMC8543043

[51] Sun Z. Association of intraplacental oxygenation patterns on dual-contrast MRI with placental abnormality and fetal brain oxygenation. Ultrasound Obstet. Gynecol. 61, 215–223 (2023). 35638228 10.1002/uog.24959 PMC9708928

[52] Aughwane R. Magnetic resonance imaging measurement of placental perfusion and oxygen saturation in early-onset fetal growth restriction. BJOG Int. J. Obstet. Gynaecol. 128, 337–345 (2021). 10.1111/1471-0528.16387 PMC7613436 32603546

[53] Zun Z., Zaharchuk G., Andescavage N. N., Donofrio M. T. & Limperopoulos C. Non-Invasive Placental Perfusion Imaging in Pregnancies Complicated by Fetal Heart Disease Using Velocity-Selective Arterial Spin Labeled MRI. Sci. Rep. 7, 16126 (2017). 29170468 10.1038/s41598-017-16461-8 PMC5700998

[54] Berning R. A. Reversed shunting across the ductus arteriosus or atrial septum in utero heralds severe congenital heart disease. J. Am. Coll. Cardiol. 27, 481–486 (1996). 8557925 10.1016/0735-1097(95)00446-7

[55] Benirschke K., Burton G. J. & Baergen R. N. Architecture of Normal Villous Trees. in Pathology of the Human Placenta (eds. Benirschke K., Burton G. J. & Baergen R. N.) 101–144 (Springer, 2012). doi:10.1007/978-3-642-23941-0_7.

[56] Castellucci M., Scheper M., Scheffen I., Celona A. & Kaufmann P. The development of the human placental villous tree. Anat. Embryol. (Berl.) 181, 117–128 (1990). 2327595 10.1007/BF00198951

[57] Demir R., Kaufmann P., Castellucci M., Erbengi T. & Kotowski A. Fetal Vasculogenesis and Angiogenesis in Human Placental Villi. Acta Anat. (Basel) 136, 190–203 (2008). 10.1159/000146886 2481376

[58] Kaufmann P., Mayhew T. M. & Charnock-Jones D. S. Aspects of Human Fetoplacental Vasculogenesis and Angiogenesis. II. Changes During Normal Pregnancy. Placenta 25, 114–126 (2004). 14972444 10.1016/j.placenta.2003.10.009

[59] Limperopoulos Brain Volume and Metabolism in Fetuses With Congenital Heart Disease. Circulation 121, 26–33 (2010). 20026783 10.1161/CIRCULATIONAHA.109.865568 PMC2819908

[60] Huen I. R1 and R2* changes in the human placenta in response to maternal oxygen challenge. Magn. Reson. Med. 70, 1427–1433 (2013). 23280967 10.1002/mrm.24581

[61] Ingram E., Morris D., Naish J., Myers J. & Johnstone E. MR Imaging Measurements of Altered Placental Oxygenation in Pregnancies Complicated by Fetal Growth Restriction. Radiology 285, 953–960 (2017). 28708473 10.1148/radiol.2017162385

[62] Siauve N. Assessment of human placental perfusion by intravoxel incoherent motion MR imaging. J. Matern. Fetal Neonatal Med. 32, 293–300 (2019). 28974131 10.1080/14767058.2017.1378334

[63] Cromb D. Assessing within-subject rates of change of placental MRI diffusion metrics in normal pregnancy. Magn. Reson. Med. 90, 1137–1150 (2023). 37183839 10.1002/mrm.29665 PMC10962570

[64] Andescavage N. N. & Limperopoulos C. Placental abnormalities in congenital heart disease. Transl. Pediatr. 10, 2148–2156 (2021). 34584887 10.21037/tp-20-347 PMC8429875

[65] Rudolph A. M. Congenital cardiovascular malformations and the fetal circulation. Arch. Dis. Child. -Fetal Neonatal Ed. 95, F132–F136 (2010). 19321508 10.1136/adc.2007.128777

[66] Josowitz R. Decreased Placental Blood Flow in Fetuses With Congenital Heart Disease is Associated With Placental Vascular Abnormalities and Impaired Fetal Growth. Circulation 146, A15837–A15837 (2022).

[67] Saini B. S. Normal human and sheep fetal vessel oxygen saturations by T2 magnetic resonance imaging. J. Physiol. 598, 3259–3281 (2020). 32372463 10.1113/JP279725

[68] Clark A. Developments in functional imaging of the placenta. Br. J. Radiol. 20211010 (2022) doi:10.1259/bjr.20211010. 35234516 PMC10321248

[69] Boyd H. A. Association Between Fetal Congenital Heart Defects and Maternal Risk of Hypertensive Disorders of Pregnancy in the Same Pregnancy and Across Pregnancies. Circulation 136, 39–48 (2017). 28424221 10.1161/CIRCULATIONAHA.116.024600

[70] Zhang S. Hypertensive Disorders in Pregnancy Are Associated With Congenital Heart Defects in Offspring: A Systematic Review and Meta-Analysis. Front. Cardiovasc. Med. 9, 842878 (2022). 35419442 10.3389/fcvm.2022.842878 PMC8995565

[71] Churchill D., Nair M., Stanworth S. J. & Knight M. The change in haemoglobin concentration between the first and third trimesters of pregnancy: a population study. BMC Pregnancy Childbirth 19, 359 (2019). 31619186 10.1186/s12884-019-2495-0 PMC6796328

[72] Giussani D. A. The fetal brain sparing response to hypoxia: physiological mechanisms. J. Physiol. 594, 1215–1230 (2016). 26496004 10.1113/JP271099 PMC4721497

[73] Ramirez Zegarra R., Dall’Asta A. & Ghi T. Mechanisms of Fetal Adaptation to Chronic Hypoxia following Placental Insufficiency: A Review. Fetal Diagn. Ther. 49, 279–292 (2022). 35760055 10.1159/000525717

[74] Benirschke K., Burton G. J. & Baergen R. N. Basic Structure of the Villous Trees. in Pathology of the Human Placenta (eds. Benirschke K., Burton G. J. & Baergen R. N.) 55–100 (Springer, 2012). doi:10.1007/978-3-642-23941-0_6.

[75] Jackson M. R., Mayhew T. M. & Boyd P. A. Quantitative description of the elaboration and maturation of villi from 10 weeks of gestation to term. Placenta 13, 357–370 (1992). 1438084 10.1016/0143-4004(92)90060-7

[76] Ewer A. K. Pulse oximetry screening for congenital heart defects in newborn infants (PulseOx): a test accuracy study. Lancet Lond. Engl. 378, 785–794 (2011). 10.1016/S0140-6736(11)60753-8 21820732

[77] Fick R. H. J., Wassermann D. & Deriche R. The Dmipy Toolbox: Diffusion MRI Multi-Compartment Modeling and Microstructure Recovery Made Easy. Front. Neuroinformatics 13, (2019). 10.3389/fninf.2019.00064 PMC6803556 31680924

[78] Seabold S. & Perktold J. Statsmodels: Econometric and Statistical Modeling with Python. Proc. 9th Python Sci. Conf. 2010, (2010).

